# Metabotype Risk Clustering Based on Metabolic Disease Biomarkers and Its Association with Metabolic Syndrome in Korean Adults: Findings from the 2016–2023 Korea National Health and Nutrition Examination Survey (KNHANES)

**DOI:** 10.3390/diseases13080239

**Published:** 2025-07-28

**Authors:** Jimi Kim

**Affiliations:** Department of Food and Nutrition, Changwon National University, Changwon 51140, Republic of Korea; jmkim206@changwon.ac.kr; Tel.: +82-55-213-3514

**Keywords:** metabotype, metabolic syndrome, biomarkers, cluster analysis, metabolic risk

## Abstract

Background: Metabolic syndrome (MetS) is a multifactorial condition involving central obesity, dyslipidemia, hypertension, and impaired glucose metabolism, significantly increasing the risk of type 2 diabetes and cardiovascular disease. Objectives: Given the clinical heterogeneity of MetS, this study aimed to identify distinct metabolic phenotypes, referred to as metabotypes, using validated biomarkers and to examine their association with MetS. Materials and Methods: A total of 1245 Korean adults aged 19–79 years were selected from the 2016–2023 Korea National Health and Nutrition Examination Survey. Metabotype risk clusters were derived using k-means clustering based on five biomarkers: body mass index (BMI), uric acid, fasting blood glucose (FBG), high-density lipoprotein cholesterol (HDLc), and non-HDL cholesterol (non-HDLc). Multivariable logistic regression was used to assess associations with MetS. Results: Three distinct metabotype risk clusters (low, intermediate, and high risk) were identified. The high-risk cluster exhibited significantly worse metabolic profiles, including elevated BMI, FBG, HbA1c, triglyceride, and reduced HDLc. The prevalence of MetS increased progressively across metabotype risk clusters (OR: 5.46, 95% CI: 2.89–10.30, *p* < 0.001). In sex-stratified analyses, the high-risk cluster was strongly associated with MetS in both men (OR: 9.22, 95% CI: 3.49–24.36, *p* < 0.001) and women (OR: 3.70, 95% CI: 1.56–8.75, *p* = 0.003), with notable sex-specific differences in lipid profiles, particularly in HDLc. Conclusion: These findings support the utility of metabotyping using routine biomarkers as a tool for early identification of high-risk individuals and the development of personalized prevention strategies in clinical and public health settings.

## 1. Introduction

Metabolic syndrome (MetS) represents a multifactorial metabolic condition defined by abdominal obesity, insulin resistance, hypertension, and dyslipidemia [1]. These interrelated risk factors markedly elevate the incidence of cardiovascular disease, type 2 diabetes, and early mortality [2]. Epidemiological studies indicate that it is associated with a 2–3 times higher risk of cardiovascular events and a more than 5-fold increased risk of type 2 diabetes, leading to elevated all-cause mortality rates [3]. The global prevalence of MetS is estimated to range between 20% and 25%, yet substantial variability exists across populations [4,5]. The variability in MetS prevalence reflects differences in ethnicity, age, gender, lifestyle factors, and the diagnostic standards applied across studies [6,7]. MetS prevalence increases with age and is generally higher among older adults [8,9]. Gender disparities have also been reported, with higher rates observed among women in certain ethnic populations [10]. The rise in MetS is largely attributed to urbanization, physical inactivity, and energy-dense dietary patterns [11]. Among the primary modifiable risk factors are obesity and sedentary lifestyles [12]. Previous studies suggest that interventions targeting weight loss and activity improvement have proven effective in reducing MetS risk [12,13].

MetS comprises a cluster of interrelated risk factors, including abdominal obesity measured by waist circumference (WC), elevated triglycerides (TG), reduced high-density lipoprotein cholesterol (HDLc), elevated blood pressure (BP), and elevated fasting blood glucose (FBG) that synergistically increase the risk of cardiovascular disease and type 2 diabetes [14]. These components often co-occur and reflect underlying disruptions in energy metabolism, insulin signaling, and adipose tissue function, suggesting a shared pathophysiological basis. Multiple diagnostic frameworks have been established by international organizations, most notably the World Health Organization (WHO), the Adult Treatment Panel III (ATP III), and the International Diabetes Federation (IDF), as shown in Supplementary Table S1 [14]. While all aim to identify individuals at heightened cardiometabolic risk, they differ in their emphasis. The WHO prioritizes insulin resistance, the ATP III emphasizes clinical practicality, and the IDF focuses on abdominal obesity, incorporating ethnicity-specific cutoffs for WC [14,15]. However, these conceptual differences have contributed to inconsistencies in MetS prevalence across populations and studies [16,17,18], highlighting the limitations of a one-size-fits-all diagnostic approach. Moreover, individuals meeting the same diagnostic criteria may exhibit diverse biological and clinical profiles due to inherent heterogeneity in metabolic regulation [19,20]. This heterogeneity underscores the need for refined stratification strategies [15]. In this study, metabotyping, an approach that classifies individuals into metabolically distinct subgroups based on biomarker patterns, offers a promising complementary tool for elucidating risk beyond conventional definitions and improving the precision of epidemiological and clinical insights.

Metabotyping refers to a classification system that stratifies individuals into metabolically homogeneous subgroups based on metabolic profiling [21,22]. This profiling typically includes biomarkers such as TG, total cholesterol, HDLc, and FBG levels [23,24]. Identification of metabotypes is commonly performed using statistical methodology such as k-means clustering [21,25]. The resulting classification can vary depending on the clustering parameters employed, including fasting serum biomarkers, dietary habits, and disease prevalence [26]. Metabotype analysis has been applied to identify groups with distinct metabolic responses to dietary interventions, thereby facilitating the development of personalized nutrition strategies. Previous studies have demonstrated that metabotypes are associated with differential dietary responses supporting their utility in personalized nutrition [23,24]. Metabotyping has been utilized in fields such as type 2 diabetes research to delineate subgroups characterized by unique patterns of disease progression and dietary responsiveness [26]. Furthermore, Dahal et al. proposed an optimized definition of metabolic types based on clinical biomarkers derived from the population-based KORA study [25]. Utilizing k-means clustering analysis, the KORA F4 cohort was stratified into three clusters [25]. Subsequently, a machine learning-based variable importance methodology was conducted to identify the most influential biomarkers for clustering. As a result, a metabolic risk disease model comprising biomarkers such as body mass index (BMI), uric acid, FBG, HDLc, and non-HDLc was developed. Statistically significant differences were observed in metabolic disease prevalence and incidence across the clusters. These metabolic types, defined using routinely measurable clinical biomarkers, hold potential for the early identification of high-risk groups and for the prevention of metabolic diseases. However, the direct applicability of these models to Asian populations remains uncertain due to inherent differences in genetics, diet, and lifestyle. Korean adults, for instance, typically consume carbohydrate-rich diets, have lower overall obesity rates but higher rates of visceral adiposity, and show unique patterns in dyslipidemia and insulin resistance. These population-specific characteristics necessitate the validation of metabotyping approaches in Korean cohorts to ensure their relevance and clinical utility.

This study aimed to identify metabotype risk clusters among Korean adults based on metabolic disease biomarkers validated in a prior study, including BMI, uric acid, FBG, HDLc, and non-HDLc. This study evaluated the characteristics of demographic, lifestyle, and metabolic profiles across the identified metabotype risk clusters to elucidate their distinguishing features. Furthermore, we investigated the association between the identified metabotype risk clusters and the prevalence of MetS and its individual components.

## 2. Materials and Methods

### 2.1. Analytical Data

The Korea National Health and Nutrition Examination Survey (KNHANES) is a nationally representative, population-based surveillance system designed to assess the health and nutritional status of the Korean population by the Korea Disease Control and Prevention Agency (KDCA), which served as the data source for the present study. The KNHANES employs a cross-sectional design and utilizes a stratified, multistage probability sampling method to collect comprehensive health data from the Korean population [27]. The survey consists of three main components, which include a health interview, a health examination, and a nutrition survey, all conducted using standardized protocols [27]. The KNHANES was conducted with approval from the Institutional Review Board (IRB) of the Korea Disease Control and Prevention Agency (KDCA) (2022-11-16-R-A), and this study was also approved in advance by the IRB of Changwon National University (7001066-202503-HR-011).

### 2.2. Study Population

**Figure 1:**
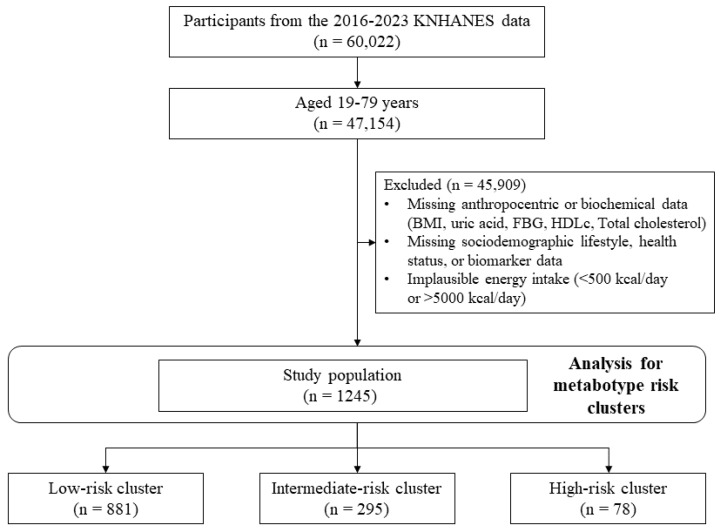
Flow chart of study subjects.

This study utilized data from the 7th to 9th cycles (2016–2023) of the KNHANES, comprising 47,154 Korean adults aged 19 to 79 years. The dataset encompassed demographic characteristics (age, sex, and sociodemographic status), health-related indicators, anthropometric measurements, and laboratory test results. Participants were excluded if they lacked data for any of the five key biomarkers used in metabotype clustering (BMI, uric acid, FBG, HDLc, and non-HDLc), as well as essential covariates including age, sex, education, occupation, alcohol consumption, smoking status, physical activity, and total energy intake. Those reporting implausible energy intakes (<500 kcal or >5000 kcal/day) were also excluded from the final analysis. Only individuals with complete data for all relevant variables were retained for analysis to avoid potential bias due to missing data. After excluding individuals with missing values for metabolic biomarkers of key covariates, a total of 1245 participants were included in the final analysis (Figure 1).

### 2.3. Biochemical and Anthropometric Parameters

Metabotypes were classified based on fasting biochemical markers, including uric acid (mg/dL), FBG (mg/dL), HDLc (mg/dL), non-HDLc (mg/dL), and TG (mg/dL), along with BMI (kg/m^2^). The metabolic biomarkers described above have been validated in prior cohort studies as optimal biomarkers for differentiating metabotypes [25]. In the present study, BMI (kg/m^2^), uric acid (mg/dL), FBG (mg/dL), HDLc (mg/dL), and non-HDLc (mg/dL) were utilized to classify metabolic disease phenotypes. Uric acid (mg/dL) levels were assessed using enzymatic colorimetric methods, while FBG (mg/dL) concentrations were determined via the ultraviolet (UV) hexokinase method, both performed on the Cobas 8000 modular analyzer (Roche Diagnostics, Mannheim, Germany). Serum total cholesterol (TC, mg/dL), HDLc (mg/dL), and TG (mg/dL) were also measured using enzymatic assays on the same platform. Non-HDLc (mg/dL) was calculated by subtracting HDLc (mg/dL) from total cholesterol. Additional biochemical parameters were evaluated as follows: glycated hemoglobin (HbA1c, %) was analyzed using high-performance liquid chromatography (HPLC) with the HLC-723G11 system (Tosoh, Tokyo, Japan); aspartate aminotransferase (AST, IU/L) and alanine aminotransferase (ALT, IU/L) levels were measured using the International Federation of Clinical Chemistry (IFCC) method on the Cobas 8000. Blood urea nitrogen (BUN, mg/dL) and creatinine (mg/dL) were assessed via kinetic colorimetric assays on the same analyzer. Hemoglobin (g/dL) concentrations were determined by cyanide-free spectrophotometry, and hematocrit (%) levels were measured using electrical impedance, both conducted on the XN-1000 automated hematology analyzer (Sysmex, Tokyo, Japan).

### 2.4. Demographic and Lifestyle Variables

Demographic information collected from participants included age, sex, education, and occupation. Education levels were grouped into less than middle school, high school, and over college. Occupational status was categorized into professional, administrative, managerial, and clerical roles; sales and service positions; employment in agriculture, manufacturing, mining, or military service; and individuals who were unemployed, engaged in housekeeping, or involved in other unspecified work. Lifestyle variables included alcohol consumption, smoking status, physical activity, and obesity according to BMI (kg/m^2^). Alcohol use was classified into non-drinker and former or current drinker. Smoking status was recorded as never smoked, former, or current smoker. Physical activity was assessed based on intensity and duration. Participants were considered physically active if they engaged in more than 75 min per week of moderate-intensity aerobic activity, or an equivalent combination of moderate- and vigorous-intensity physical activity. BMI (kg/m^2^) was calculated from measured height and weight, and categorized into underweight (<18.5 kg/m^2^), normal weight (18.5–22.9 kg/m^2^), overweight (23–24.9 kg/m^2^), and obese (≥25 kg/m^2^), according to WHO classification criteria.

### 2.5. MetS Classification and Diagnosis

The classification and diagnosis of MetS were conducted in accordance with harmonized international criteria incorporating WC cutoffs specific to the Korean population [18,28]. Participants were identified as having MetS if they exhibited at least three of the following five risk factors: (1) elevated WC with cutoff points specific to South Koreans (≥90 cm in men and ≥85 cm in women); (2) elevated TG levels (≥150 mg/dL or specific treatment for lipid abnormality); (3) reduced HDLc level (<40 mg/dL in men and <50 mg/dL in women or drug treatment for lipid abnormality); (4) elevated BP (systolic BP, ≥130 mmHg and diastolic BP, ≥85 mmHg), or treatment for diagnosed hypertension; (5) elevated FBG level (≥100 mg/dL or diagnosed type 2 diabetes).

### 2.6. Metabotype Risk Clusters

Clustering was conducted using the k-means algorithm, as implemented in IBM SPSS Statistics (version 25.0). Prior to clustering, all continuous variables were standardized using z-scores to ensure equal weighting during distance calculations, as the biomarkers used (BMI, uric acid, FBG, HDLc, and non-HDLc) vary in scale and units. Although TG exhibited a right-skewed distribution, it was excluded from the clustering variables due to its distributional characteristics and potential to distort centroid-based algorithms such as k-means. The optimal number of clusters was determined using the elbow method based on within-cluster sum of squares (WCSS) and supported by the Calinski–Harabasz index (Supplementary Figure S1). The elbow plot demonstrated a clear inflection point at k = 3, and the Calinski–Harabasz index was highest for the 3-cluster solution (182.8), indicating good separation between clusters. Although the average silhouette score was moderate (0.186), the resulting clusters were clinically interpretable and aligned with known metabolic phenotypes (Supplementary Figure S2). TG and HbA1c were not included in the initial clustering model despite their metabolic relevance, to maintain focus on biomarkers that are both routinely collected and less variable across populations. This decision aligns with prior studies that emphasized simplicity and clinical accessibility in cluster construction [25].

### 2.7. Statistical Analysis

The analysis utilized data from the NHANES, which utilizes a multistage, complex survey design. For comparisons between men and women, continuous variables were summarized as means with standard errors (SEs) and analyzed using complex samples *t*-tests, while categorical variables were presented as frequencies and percentages and evaluated using complex samples chi-square tests. To examine differences across the three identified metabotype risk clusters, continuous variables were assessed using a generalized linear model adjusted for stratification variables, and categorical variables were analyzed using complex samples chi-square tests for complex survey data. Multivariable logistic regression was used to estimate odds ratios (ORs) and their corresponding 95% confidence intervals (CIs). The multivariable models were adjusted for sex, age, alcohol consumption, smoking status, physical activity (both vigorous and moderate intensity), and total energy intake. Statistical analyses were conducted using SAS software, version 9.4 (SAS Institute Inc., Cary, NC, USA). A two-tailed *p*-value of less than 0.05 was considered indicative of statistical significance. A post hoc power analysis based on the observed effect size (Cohen’s f) and the total sample size demonstrated sufficient statistical power (greater than 0.99) to detect significant differences among the metabotype risk clusters at a significance level of 0.05.

## 3. Results

### 3.1. Characteristics of the Study Population

Table 1 summarizes participant characteristics by gender, including sociodemographic characteristics, lifestyle factors, and MetS components. The mean age was 64.2 ± 0.4 years, with women being significantly older than men (*p* < 0.001). Education and occupation differed significantly by gender (*p* < 0.001). With regard to health-related behaviors, men had significantly higher rates of current and past alcohol consumption and smoking compared to women (*p* < 0.001). Men also reported more participation in vigorous-intensity (*p* = 0.003) and moderate-intensity physical activity (*p* < 0.001). Among the total participants, 93.9% reported using antihypertensive medications, with a higher proportion observed in women than in men (*p* = 0.046). Lipid-lowering medication use was reported by 87.3% of the participants, with significantly higher usage in women compared to men (*p* = 0.002). The proportion of participants using antidiabetic medications was 95.0%, with no significant gender difference. Men had significantly higher total energy intake than women (*p* < 0.001). The overall prevalence of obesity did not differ significantly between genders, but men had a slightly higher prevalence. The prevalence of MetS was significantly higher in women than in men (*p* = 0.013). Among the individual components of MetS, WC (*p* < 0.001) and TG (*p* = 0.004) levels were significantly higher in men. On the other hand, HDLc levels were significantly higher in women (*p* < 0.001). BP measurements also showed differences by gender. Women had higher SBP (*p* < 0.001), and men had higher DBP (*p* = 0.017). Fasting blood glucose levels were similar between genders, and no significant differences were observed.

### 3.2. Sociodemographic and Lifestyle Characteristics Across Metabotype Risk Clusters

Table 2 presents the distribution of sociodemographic and lifestyle characteristics across the three metabotype risk clusters identified via k-means clustering: low-risk (*n* = 881), intermediate-risk (*n* = 295), and high-risk (*n* = 78). The principal component analysis (PCA) clearly differentiates the clusters based on biomarkers (BMI, uric acid, FBG, HDLc, and non-HDLc) patterns, supporting the validity of the clustering approach (Figure S1). PCA was conducted using the five clustering biomarkers to provide a two-dimensional visualization of cluster separation. As shown in Supplementary Figure S2, the first two principal components (PC1 and PC2) explained 26.6% and 21.8% of the total variance, and the identified clusters formed visibly distinct groupings in the PCA space. This confirms the multidimensional nature of the cluster separation and supports the validity of the k-means classification. High-risk participants were significantly younger than those in the other groups (*p* < 0.001). While the proportion of males was slightly higher in the high-risk group, the difference was not significant. Educational level and occupation showed no significant differences across clusters, though sales and service occupations increased with risk level. All three clusters had similar alcohol consumption and smoking status. No significant difference was observed in the physical activity patterns between the clusters. Antihypertensive medication use was slightly lower in the intermediate-risk cluster compared to the low-risk and high-risk clusters, although the difference did not reach statistical significance. Lipid-lowering medications were used by 92.3% of individuals in the low-risk cluster, but the proportion significantly declined in the intermediate-risk (74.6%) and high-risk (79.5%) clusters (*p* < 0.001). Antidiabetic medication use showed a significant increasing trend across the clusters, from 90.5% in the intermediate-risk group to 98.7% in the high-risk group (*p* < 0.001), with the low-risk group at 96.1%. Obesity was more prevalent in the intermediate-risk (54.5%) and high-risk (54.0%) clusters than in the low-risk cluster (49.0%), with a corresponding decline in normal-weight individuals as risk increased. Although total energy intake was highest in the high-risk cluster compared to the other groups, the difference was not statistically significant. Differences across metabotype risk clusters remained largely consistent in analyses stratified by sex (Supplementary Tables S2–S5). In both men and women, higher-risk clusters were associated with younger age (men, *p* = 0.004; women, *p* < 0.001) and differential patterns in health behaviors and medication use. Among men, smoking prevalence was lower in the high-risk cluster (*p* = 0.003), while alcohol consumption differences were modest (*p* = 0.031). For women, occupation categories differed across clusters (*p* = 0.018), and the use of antihypertensive (*p* = 0.003), lipid-lowering (*p* < 0.001), and antidiabetic medications (*p* = 0.001) was more prevalent in higher-risk clusters (Supplementary Table S2).

### 3.3. Comparison of Metabolic Biomarkers Across Metabotype Risk Clusters

Table 3 presents a comparative analysis of metabolic biomarkers across the three identified metabotype risk clusters. Several biomarkers used for clustering and additional clinical parameters exhibited statistically significant differences among the clusters. Among the biomarkers used for clustering, participants in the high-risk cluster had significantly higher BMI relative to those in the low- and intermediate-risk clusters (*p* = 0.002). Uric acid levels were significantly higher in the intermediate-risk cluster compared to both the low- and high-risk clusters (*p* = 0.008). FBG levels increased progressively across all clusters with a highly significant trend (*p* < 0.001). HDLc levels in the high-risk cluster were significantly lower than in the other clusters (*p* = 0.039). In contrast, non-HDLc levels were highest in the intermediate-risk cluster (*p* < 0.001). Clear metabolic contrasts were observed across the three identified clusters. Cluster 3 exhibited markedly elevated FBG and moderately reduced HDLc, suggestive of a dysglycemic and atherogenic phenotype. The largest effect sizes were observed for non-HDLc (f = 0.75) and FBG (f = 0.73), indicating substantial between-group differences. In contrast, BMI and uric acid showed smaller effect sizes (f ≈ 0.11), suggesting a less prominent role in discriminating clusters. Regarding additional clinical parameters, HbA1c levels increased significantly with increasing metabolic risk (*p* < 0.001). Similar patterns were observed for TC, TG, and hemoglobin levels with statistically significant differences across all clusters (*p* < 0.001). Hematocrit levels also increased significantly with increasing metabolic risk (*p* = 0.001). In contrast, no significant differences were observed for hepatic enzymes (AST, ALT), renal function markers (BUN, creatinine), or several hematological parameters between the three clusters. Metabolic biomarkers showed consistent gradients across risk clusters in both sexes (Supplementary Table S3). BMI increased significantly across clusters (men, *p* = 0.044; women, *p* = 0.025). FBG and HbA1c were markedly elevated in high-risk clusters both in men (FBG, *p* < 0.001; HbA1c, *p* < 0.001) and women (FBG, *p* < 0.001; HbA1c, *p* < 0.001). Non-HDLc and triglycerides also increased significantly with cluster risk (all *p* < 0.001). HDLc was significantly lower in men (*p* = 0.040) but did not differ significantly in women. Uric acid levels increased across clusters in women (*p* < 0.001) but not in men.

### 3.4. Comparison of MetS Components Among Metabotype Risk Clusters

Table 4 presents the distribution of MetS components across the three metabotype risk clusters. Statistically significant differences were observed in all individual MetS components with a consistent trend indicating deteriorating metabolic profiles in the higher-risk cluster. WC increased progressively across the clusters (*p* < 0.001). TG levels were markedly elevated in the high-risk cluster compared to the low-risk group (*p* < 0.001). Conversely, HDLc levels declined with increasing cluster metabolic risk, reaching the lowest values in the high-risk cluster (*p* = 0.039). BP also showed a significant upward trend across clusters. SBP was highest in the high-risk cluster compared to the low-risk cluster (*p* = 0.002), while DBP also trended upward. FBG exhibited the most marked difference, nearly doubling in the high-risk cluster compared to the low-risk cluster (*p* < 0.001). The prevalence of MetS increased significantly across clusters with 49.3% in the low-risk, 70.9% in the intermediate-risk, and 84.6% in the high-risk cluster (*p* < 0.001). Furthermore, the number of MetS components met by individuals also differed significantly among clusters. In the high-risk cluster, 19.2% of participants met all five diagnostic criteria, compared to 9.5% in the intermediate-risk and only 4.4% in the low-risk cluster (*p* < 0.001). The proportions of individuals meeting exactly three or four components of MetS also increased with risk level. Components of metabolic syndrome varied by cluster within each sex (Supplementary Table S4). WC increased with cluster risk in women (*p* = 0.008). TG and FBG showed strong increasing trends (all *p* < 0.001). SBP and DBP increased significantly across clusters in men (*p* = 0.005 and *p* < 0.001, respectively). The prevalence of MetS rose markedly across clusters (all *p* < 0.001), with the number of MetS components also increasing (men, *p* = 0.009; women, *p* = 0.006).

### 3.5. Association Between Metabotype Risk Clusters and MetS

Table 5 presents the associations between metabolic risk clusters and the presence of MetS along with its individual components. Compared to the low-risk cluster, those in the intermediate-risk cluster had a significantly higher likelihood of having MetS (OR: 2.43, 95% CI: 1.82–3.23, *p* < 0.001). The high-risk cluster demonstrated the greatest risk of MetS (OR: 5.46, 95% CI: 2.89–10.30, *p* < 0.001). Among the individual MetS components, increased WC was significantly associated with the high-risk cluster (OR: 2.19, 95% CI: 1.24–3.88, *p* = 0.007), but no significant association was observed in the intermediate-risk cluster. For elevated TG, the risk was significantly increased in both the intermediate-risk cluster (OR: 4.38, 95% CI: 3.30–5.81, *p* < 0.001) and the high-risk cluster (OR: 4.84, 95% CI: 2.95–7.93, *p* < 0.001). Reduced HDLc levels were significantly associated with the high-risk cluster (OR: 2.24, 95% CI: 1.37–3.65, *p* = 0.001), but were not statistically significant in the intermediate-risk cluster. Elevated BP showed a significant association with the intermediate-risk cluster (OR: 1.42, 95% CI: 1.09–1.86, *p* = 0.010), whereas no significant association was found in the high-risk cluster. In the high-risk cluster, FBG levels were elevated in all participants (100.0%), leading to complete separation and precluding the estimation of the ORs. In contrast, no significant difference in FBG elevation was observed between the intermediate- and low-risk clusters. Adjusted logistic regression analyses confirmed significantly higher ORs of MetS in the high-risk cluster compared to the low-risk cluster (men, OR: 9.22, 95% CI: 3.49–24.36, *p* < 0.001; women, OR: 3.70, 95% CI: 1.56–8.75, *p* = 0.003) (Supplementary Table S5). Elevated TG were strongly associated with higher-risk clusters in both sexes (men, OR: 5.80, 95% CI: 2.87–11.74; women, OR: 4.16, 95% CI: 2.05–8.44; both *p* < 0.001). Reduced HDLc showed a significant association in men only (OR: 3.27, 95% CI: 1.67–6.42, *p* < 0.001). Elevated BP was associated with risk clusters in men (OR: 2.17, 95% CI: 1.12–4.19, *p* = 0.021) but not in women. Associations with elevated WC in women were borderline significant.

## 4. Discussion

Three distinct metabotype risk groups of low-risk, intermediate-risk, and high-risk were identified among Korean adults based on validated metabolic disease biomarkers, including BMI, uric acid, FBG, HDLc, and non-HDLc. Individuals classified into the high-risk cluster exhibited significantly adverse metabolic profiles characterized by elevated BMI, FBG, and TG alongside reduced HDLc level compared with those in the low-risk cluster. The prevalence of MetS and its components increased progressively across higher metabotype risk clusters. Moreover, high-risk cluster demonstrated a substantially greater likelihood of MetS driven by elevated TG, central obesity, and impaired glycemic control.

Metabotyping enables stratification of individuals at differential cardiometabolic risk using routinely collected biomarkers, supporting targeted prevention efforts [29,30,31]. Metabotype classification and chronic disease risk have been investigated in European and American populations, guided by recommendations from the European Society of Cardiology (ESC) and the American Heart Association (AHA) [32]. Using data from the MARE consortium, which includes participants from 10 European countries, various clusters of metabolic syndrome components were identified and found to be associated with cardiovascular disease risk [32]. A subsequent MARE consortium study further demonstrated that specific metabolic syndrome clusters were linked to increased arterial stiffness in both European and American populations [33]. In the present study, three distinct metabolic types (low-risk, intermediate-risk, and high-risk) were delineated among Korean adults based on the availability and clinical relevance of selected metabolic disease biomarkers. Participants in the high-risk cluster demonstrated substantially more adverse metabolic profiles, particularly regarding glycemic regulation (FBG and HbA1c), lipid parameters, and BMI, thereby affirming the clinical validity of the clustering model. These metabolic alterations, particularly elevated BMI, TG, and impaired glycemic control, are known to be associated with dysregulation of adipokines, which play key roles in mediating chronic low-grade inflammation and insulin resistance in MetS [34]. Although adipokine levels were not measured in the current study, the biomarker profile may reflect underlying inflammation. These findings are consistent with prior studies highlighting dyslipidemia (elevated TG and reduced HDLc) and impaired glycemic control as critical features of the high-risk metabolic subgroup [21,22]. Among the biomarkers used in clustering, uric acid has garnered increasing attention as a pathogenic contributor to metabolic dysfunction. Elevated uric acid levels have been implicated in the induction of oxidative stress, activation of pro-inflammatory pathways, and suppression of endothelial nitric oxide bioavailability. These mechanisms collectively promote insulin resistance and vascular inflammation, both of which are hallmark features of metabolic syndrome. In this study, the inclusion of uric acid in the clustering model not only enhanced the discriminatory accuracy but also added biological plausibility, as elevated uric acid levels may partially explain the metabolic divergence observed among the identified clusters, particularly the high-risk group. These findings align with previous literature underscoring the pathophysiological relevance of uric acid in cardiometabolic disease progression [35,36].

An unexpected finding of this study was that individuals in the high-risk metabotype cluster were, on average, younger than those in the intermediate-risk and low-risk clusters. This result contrasts with much of the existing literature, which typically reports increased metabolic risk with advancing age due to cumulative exposure to lifestyle risk factors, progressive insulin resistance, and age-related physiological decline [9]. However, several potential explanations may account for this observation in this study among the Korean population. First, younger adults in Korea may increasingly exhibit unhealthy lifestyle patterns such as high consumption of energy-dense diets, sedentary behavior, and reduced physical activity, leading to the earlier onset of metabolic dysregulation. Second, the KNHANES survey design includes cross-sectional sampling that may capture cohort effects. The older adults who survived to participate may represent a healthier subset due to survival bias, potentially underestimating the true metabolic risk in older groups. Third, the clustering approach in this study was based purely on biomarker profiles standardized across age groups, which may accentuate metabolic vulnerability that is independent of chronological age. Lastly, the relatively high prevalence of central obesity and visceral adiposity even in younger East Asian adults may contribute to this age-independent clustering of metabolic risk. These possibilities highlight the need for further longitudinal research to clarify whether this pattern reflects a true epidemiological shift toward earlier metabolic syndrome onset or methodological artifacts of cross-sectional survey design.

Recent studies show that lifestyle-based clusters, such as those defined by physical activity or diet, often fail to align consistently with distinct metabolic profiles [37,38,39]. In contrast, metabolic parameters exhibit more stable and robust clustering across lifestyle groupings, highlighting the value of biomarker-based approaches [40,41]. Population-based studies, including those in China and the NHANES data, found weak alignment between lifestyle classifications and metabolic traits, while pediatric cohorts showed stronger associations in certain contexts [38,39,42]. In our study, although metabolic risk differed across clusters, there were no significant differences in conventional lifestyle factors like alcohol use, smoking, or physical activity. This suggests that biomarker-driven metabotype clustering may offer a more precise way to identify high-risk groups, revealing early-onset metabolic vulnerability that is independent of age [43]. This finding supports the notion that biomarker-driven, metabolically-defined clusters may provide a more precise framework for identifying high-risk populations than lifestyle-based classification alone. Moreover, the observation that the high-risk metabotype cluster was younger than the low-risk and intermediate-risk clusters suggests a pattern of early-onset metabolic dysregulation. This dissociation from age-related trends implies the existence of intrinsic metabolic vulnerability, independent of the aging process.

The high prevalence of MetS and its individual components within the high-risk metabotype cluster underscores the utility of cluster-based approaches in effectively stratifying and predicting MetS risk. In this study, the three clusters were differentiated by stepwise increases in cardiometabolic biomarkers, indicating a gradient of metabolic risk. The biomarkers of WC, TG, FBG, and BP significantly increased with higher risk levels, while HDLc demonstrated a corresponding decline. The prevalence of MetS was notably elevated across the low-risk (49.3%), intermediate-risk (70.9%), and high-risk (84.6%) clusters. Furthermore, the proportion of individuals meeting all diagnostic criteria for MetS was highest in the high-risk cluster, and the number of diagnostic components present increased in parallel with risk stratification. High-risk cluster exhibited a metabolic profile consistent with features of insulin resistance and dysregulated lipid metabolism. The simultaneous elevation of TG and FBG, coupled with central obesity, is suggestive of potential mechanisms such as increased hepatic lipogenesis, reduced insulin sensitivity in adipose tissue, and impaired glucose uptake in peripheral tissues [44]. These pathophysiological features are consistent with the progression toward type 2 diabetes and cardiovascular disease risk, reinforcing the clinical significance of this cluster. Hypertriglyceridemia is a hallmark of MetS and is intricately associated with other core components, including central obesity, hypertension, and hyperglycemia [6]. Elevated TG levels are associated with insulin resistance, a key pathophysiological feature of MetS [45]. In addition, hypertriglyceridemia facilitates the formation of small dense low-density lipoprotein (LDL) particles, which exhibit enhanced atherogenic potential [46]. The TG-to-HDLc ratio has emerged as a robust predictor of MetS and its associated complications [47]. A higher TG-to-HDLc ratio has been consistently linked to increased risks of insulin resistance, atherosclerosis, and overall cardiometabolic diseases [47]. Low HDLc levels indicate impaired lipid clearance and are a recognized marker of dyslipidemia [48]. Notably, HDLc concentrations are influenced by both sex and ethnicity, which may contribute to population-level differences in the prevalence and expression of MetS [49]. The co-occurrence of elevated TG levels and reduced HDLc is recognized as a strong marker of cardiovascular disease risk [50]. Prior studies have linked elevated TG-to-HDLc ratios to increased cardiovascular disease risk and hepatic fat accumulation, particularly in younger adults and those with visceral obesity [51,52]. This ratio may therefore serve as a simple yet powerful indicator of cardiometabolic risk in population-level screening.

The differences in FBG, HbA1c, hemoglobin, and hematocrit levels across metabotype risk clusters reflect key aspects of metabolic dysfunction and may serve as early hematological markers of metabolic risk. FBG indicates short-term glycemic status while HbA1c reflects long-term control, making both critical for identifying individuals at elevated risk of MetS [53]. Elevated FBG characterizes impaired fasting glucose (IFG), a prediabetic state with a high prevalence of MetS, underscoring its clinical relevance in early risk stratification [54,55]. HbA1c has been shown to correlate with various MetS components, including increased waist circumference, elevated BP, and hypertriglyceridemia, highlighting its value as a comprehensive risk marker [56]. Using both FBG and HbA1c together provides a more nuanced and integrated assessment of metabolic health and emphasizes the importance of glycemic management in reducing cardiometabolic complications [57]. This dual-marker approach improves the prediction and management of MetS [58,59]. In these findings, a comprehensive management strategy that integrates glycemic markers with the full spectrum of MetS components is essential for effective prevention and intervention efforts aimed at reducing long-term complications [60]. In addition to the overall analysis, sex-stratified analyses were conducted to examine differences in metabotype clustering and associated metabolic characteristics. HDLc showed a significant decreasing trend across clusters in men but remained stable in women, while WC increased significantly in both sexes with varying effect sizes. These results indicate that although the overall metabotype framework applies to both sexes, specific metabolic traits may contribute differently to cluster membership and disease risk. Such sex-specific patterns highlight the importance of incorporating gender considerations into clinical risk assessment and tailoring prevention strategies.

The strengths of this study are presented as follows. First, the data used in this study are nationally representative population data using the KNHANES based on a cross-sectional design and stratified multistage probability sampling. The reliability of the results can be increased by utilizing the KNHANES database with high data quality collected using a standardized protocol. Second, this study employed biomarkers with established clinical and biological validity as demonstrated in previous studies, thereby ensuring the reproducibility and reliability of the findings. In this study, participants were classified into three distinct metabotype risk clusters based on metabolic biomarker characteristics, revealing significant and clinically meaningful metabolic patterns across clusters, which underscores the potential for clinical application of these validated biomarkers. Third, a standardized clustering methodology was applied utilizing k-means analysis in conjunction with z-score normalization. This approach facilitated consistency and comparability across variables, thereby minimizing bias due to variable scaling and improving the validity of the cluster identification.

This study has several limitations that should be considered. First, the cross-sectional design of this study precludes causal inference. All observed associations should be interpreted as correlations rather than causal relationships. The temporal ordering of biomarker changes and disease onset cannot be determined, and potential reverse causality cannot be ruled out. These findings should be viewed as hypothesis-generating and warrant longitudinal confirmation. Moreover, the possibility of reverse causality cannot be ruled out, particularly in younger individuals with unexpectedly high-risk profiles. In older participants, survivor bias may have led to underrepresentation of individuals with severe metabolic impairment, potentially attenuating cluster differences. Additionally, as WC and HDLc distributions differ by sex and age, future clustering analyses should consider sex-stratified or age-adjusted models to enhance precision and clinical relevance. Second, the exclusion of genetic, dietary intake, and gut microbiome data may have resulted in residual confounding. These unmeasured variables could influence biomarker levels such as uric acid or HDLc independently of other metabolic factors, potentially masking or exaggerating the distinctiveness of certain clusters. While key sociodemographic and lifestyle factors were adjusted, important sources of unmeasured confounding, such as insulin levels, inflammatory markers (e.g., CRP, IL-6), and genetic susceptibility, were not included. These factors could influence both biomarker clustering and disease expression, potentially altering the observed metabotype profiles. Additionally, future studies integrating these dimensions could elucidate biological mechanisms driving metabolic heterogeneity. Third, although the findings are based on a nationally representative Korean sample, the lack of external validation in diverse populations limits their generalizability. Biomarker distributions and their disease associations can vary substantially across ethnic, regional, and dietary contexts. For example, baseline HDLc and uric acid levels differ across East Asian, Western, and South Asian populations, affecting the clustering structure. To establish broader applicability, future research should replicate this clustering framework in multiethnic cohorts with differing metabolic baselines. Lastly, the metabotyping approach was not assessed for predictive accuracy. Longitudinal validation in prospective cohorts is needed to determine whether metabotype risk clusters can predict incident metabolic syndrome or cardiovascular outcomes more effectively than existing MetS definitions.

Our findings suggest that the routine use of clinical biomarkers to identify metabolic risk clusters could optimize screening programs for Korean adults, leading to more effective clinical practices and public health initiatives. Early identification of individuals at high risk of MetS offers opportunities for targeted interventions, including personalized lifestyle and nutrition counseling and early pharmacological management. Integrating metabotype risk clustering into health screening programs could strengthen risk prediction models, contributing to the realization of precision medicine for the prevention of metabolic and cardiovascular diseases.

In conclusion, this study identified three distinct metabotype risk clusters among Korean adults using five routinely measurable clinical biomarkers (BMI, uric acid, FBG, HDLc, and non-HDLc). Individuals in the high-risk metabotype cluster exhibited a convergence of dysglycemia, dyslipidemia, and obesity, indicating a consolidated profile of early cardiometabolic dysfunction. Unlike conventional MetS criteria that function as binary thresholds, metabotyping captures underlying biological heterogeneity and enables continuous risk stratification. The novel contribution of this study lies in demonstrating that metabotype-based clustering not only aligns with but also refines MetS diagnosis by identifying high-risk individuals who may be overlooked by existing definitions. This approach could be integrated into clinical workflows using routinely available biomarkers, offering an accessible method for population-wide screening. For instance, a patient in their 30s with a borderline FBG and normal BP but elevated uric acid and low HDLc may not meet MetS criteria but would be classified into the high-risk cluster, triggering early intervention. Furthermore, sex-stratified analyses indicated that while the overall metabotype framework was applicable to both sexes, specific metabolic traits contributed differently to cluster characteristics and disease risk. HDLc levels declined significantly across clusters in men but remained relatively stable in women, whereas WC increased in both sexes with varying effect sizes. These findings support the inclusion of sex-specific considerations in the application of metabotype-based screening strategies for more precise risk stratification and targeted intervention. Compared to conventional MetS checklists, metabotyping provides dynamic insights into metabolic trajectory, informing personalized nutrition plans or early pharmacologic intervention before full syndrome onset. This stratification approach may help inform hypothesis generation for precision prevention strategies, though longitudinal validation is needed before recommending changes in health policies.

## Figures and Tables

**Table 1:** Characteristics of the study population on the Korea National Health and Nutrition Examination Survey (KNHANES) 2016–2023.

	Total (*n* = 1245)	Men (*n* = 571)	Women (*n* = 683)	*p*-Value
Age (years)	64.2 ± 0.4	62.5 ± 0.6	66.1 ± 0.4	<0.001
Education (*n*, %)				
Less than middle school	735 (52.1)	245 (38.2)	490 (66.9)	<0.001
High school	329 (27.8)	195 (33.3)	134 (22.0)	
Over college	190 (20.1)	131 (28.5)	59 (11.1)	
Occupation (*n*, %)				
Professionals, administrative, management, office jobs	109 (11.5)	81 (17.3)	28 (5.2)	<0.001
Sales and service positions	100 (9.2)	34 (7.7)	66 (10.8)	
Agriculture, manufacturing, mining, army service	332 (25.1)	198 (33.3)	134 (17.3)	
Housekeeping, unemployment, and others	767 (52.6)	258 (42.7)	455 (66.7)	
Alcohol consumption (*n*, %)				
Past or current	1025 (84.0)	547 (95.7)	478 (71.4)	<0.001
Never	229 (16.0)	24 (4.3)	205 (28.6)	
Smoking (*n*, %)				
Past or current	546 (47.8)	480 (84.2)	66 (8.9)	<0.001
Never	708 (52.2)	91 (15.8)	617 (91.1)	
Vigorous intensity of physical activity (*n*, %)				
Yes	29 (2.91)	23 (4.5)	6 (1.2)	<0.003
No	1225 (97.1)	548 (95.5)	677 (98.8)	
Moderate intensity of physical activity (*n*, %)				
Yes	209 (19.1)	123 (24.5)	86 (13.3)	<0.001
No	1045 (80.9)	448 (75.5)	597 (86.7)	
Antihypertensive medication use (*n*, %)				
Yes	1178 (93.9)	528 (92.5)	650 (95.2)	0.046
No	76 (6.1)	43 (7.5)	33 (4.8)	
Lipid-lowering medication use (*n*, %)				
Yes	1095 (87.3)	480 (84.1)	615 (90.0)	0.002
No	159 (12.7)	91 (15.9)	68 (10.0)	
Antidiabetic medication use (*n*, %)				
Yes	1191 (95.0)	546 (95.6)	645 (94.4)	0.34
No	63 (5.0)	25 (4.38)	38 (5.56)	
Obesity (*n*, %)				
Underweight	8 (0.4)	4 (0.5)	4 (0.4)	0.43
Normal	281 (20.8)	122 (19.4)	159 (22.2)	
Pre-obese	356 (28.2)	159 (27.2)	197 (29.2)	
Obese	609 (50.7)	286 (53.0)	323 (48.2)	
Total energy intake (kcal/day)	1749.31 ± 32.12	2019.36 ± 55.03	1460.32 ± 23.94	<0.001
MetS (*n*, %)				
Yes	709 (56.5)	301 (52.7)	408 (59.7)	0.013
No	545 (43.5)	270 (47.3)	275 (40.3)	
Parameters of MetS				
WC (cm)	90.57 ± 9.23	93.09 ± 9.03	88.46 ± 8.86	<0.001
TG (mg/dL)	144.04 ± 87.63	151.96 ± 98.48	137.41 ± 76.85	0.004
HDLc (mg/dL)	48.42 ± 12.45	46.33 ± 12.53	50.17 ± 12.13	<0.001
SBP (mmHg)	127.57 ± 15.50	125.97 ± 14.37	128.91 ± 16.27	<0.001
DBP (mmHg)	73.53 ± 9.21	74.22 ± 9.66	72.96 ± 8.79	0.017
FBG (mg/dL)	131.75 ± 36.97	133.23 ± 37.89	130.52 ± 36.16	0.20

DBP, diastolic blood pressure; FBG, fasting blood glucose; HDLc, high-density lipoprotein cholesterol; SBP, systolic blood pressure; TG, triglycerides; WC, waist circumference. Values for categorical variables are shown as number and percentage (*n*, %), and continuous variables as mean ± SE, adjusted for the complex sampling design. A *p*-value below 0.05 was considered statistically significant.

**Table 2:** Sociodemographic and lifestyle characteristics across metabotype risk clusters.

	Metabotype Risk Clusters			
	Low-Risk	Intermediate-Risk	High-Risk	*p*-Value
N	881	295	78	
Age (years)	65.42 ± 0.46	62.39 ± 0.69	58.88 ± 1.45	<0.001
Sex (*n*, %)				
Men	398 (45.2)	131 (44.4)	42 (53.9)	0.31
Women	483 (54.8)	164 (55.6)	36 (46.2)	
Education (*n*, %)				
Less than middle school	538 (54.7)	155 (46.6)	42 (44.9)	0.08
High school	220 (27.1)	84 (27.4)	25 (37.1)	
Over college	123 (18.2)	56 (26.0)	11 (18.0)	
Occupation (*n*, %)				
Professionals, administrative, management, office jobs	72 (11.0)	30 (13.6)	7 (9.2)	0.32
Sales and service positions	62 (8.0)	27 (10.6)	11 (16.5)	
Agriculture, manufacturing, mining, army service	244 (25.3)	69 (23.7)	19 (27.3)	
Housekeeping, unemployment, and others	503 (55.8)	169 (52.1)	41 (47.0)	
Alcohol consumption (*n*, %)				
Past or current	719 (83.7)	240 (82.8)	66 (90.7)	0.24
Never	162 (16.3)	55 (17.2)	12 (9.3)	
Smoking (*n*, %)				
Past or current	375 (47.4)	134(50.4)	37 (43.7)	0.59
Never	506 (52.7)	161 (49.6)	41 (56.3)	
Vigorous intensity of physical activity (*n*, %)				
Yes	17 (2.4)	11 (4.7)	1 (1.7)	0.24
No	864 (97.6)	284 (95.3)	77 (98.3)	
Moderate intensity of physical activity (*n*, %)				
Yes	144 (18.3)	55 (21.1)	10 (20.0)	0.71
No	737 (81.7)	240 (78.9)	68 (80.0)	
Antihypertensive medication use (*n*, %)				
Yes	836 (94.9)	269 (91.2)	73 (93.6)	0.07
No	45 (5.1)	26 (8.8)	5 (6.4)	
Lipid-lowering medication use (*n*, %)				
Yes	813 (92.3)	220 (74.6)	62 (79.5)	<0.001
No	68 (7.7)	75 (25.4)	16 (20.5)	
Antidiabetic medication use (*n*, %)				
Yes	847 (96.1)	267 (90.5)	77 (98.7)	<0.001
No	34 (3.9)	28 (9.5)	1 (1.3)	
Obesity (*n*, %)				
Underweight	8 (0.6)	0 (0)	0 (0)	0.05
Normal	210 (23.2)	60 (17.1)	11 (9.6)	
Pre-obese	245 (27.3)	84 (28.4)	27 (36.4)	
Obese	418 (49.0)	151 (54.5)	40 (54.0)	
Total energy intake (kcal/day)	1708.23 ± 36.62	1745.47 ± 50.88	2066.94 ± 211.06	0.26

Values for categorical variables are shown as number and percentage (*n*, %), and continuous variables as mean ± SE, adjusted for the complex sampling design. A *p*-value below 0.05 was considered statistically significant.

**Table 3:** Comparison of metabolic biomarkers across metabotype risk clusters.

	Metabotype Risk Clusters			
	Low-Risk	Intermediate-Risk	High-Risk	*p*-Value
Biomarkers used for metabotype clusters				
BMI (kg/m^2^)	25.67 ± 0.16	26.47 ± 0.24	27.19 ± 0.54	0.002
Uric acid (mg/dL)	5.03 ± 0.06	5.37 ± 0.09	5.13 ± 0.20	0.008
FBG (mg/dL)	121.97 ± 0.81	133.15 ± 1.75	235.00 ± 5.83	<0.001
HDLc (mg/dL)	48.43 ± 0.46	46.93 ± 0.78	44.45 ± 1.76	0.039
Non-HDLc (mg/dL)	92.04 ± 0.71	154.45 ± 1.91	119.52 ± 5.38	<0.001
Other biomarkers				
HbA1c (%)	6.81 ± 0.03	7.17 ± 0.08	9.67 ± 0.23	<0.001
TC (mg/dL)	140.49 ± 0.81	201.41 ± 2.18	164.00 ± 5.40	<0.001
TG (mg/dL)	122.14 ± 2.15	206.02 ± 8.90	215.78 ± 20.38	<0.001
AST (IU/L)	26.18 ± 0.43	26.60 ± 0.95	27.74 ± 2.02	0.71
ALT (IU/L)	26.35 ± 0.66	26.74 ± 1.14	31.92 ± 2.77	0.15
BUN (mg/dL)	17.11 ± 0.21	17.15 ± 0.48	17.14 ± 0.69	0.99
Creatinine (mg/dL)	1.03 ± 0.01	1.07 ± 0.03	1.02 ± 0.02	0.42
Hemoglobin (g/dL)	13.47 ± 0.06	14.05 ± 0.10	14.53 ± 0.20	<0.001
Hematocrit (%)	41.58 ± 0.18	42.51 ± 0.27	43.33 ± 0.53	0.001

ALT, alanine aminotransferase; AST, aspartate aminotransferase; BMI, body mass index; BUN, blood urea nitrogen; FBG, fasting blood glucose; HbA1c, hemoglobin A1c; HDLc, high-density lipoprotein cholesterol; Non-HDLc, non-high-density lipoprotein cholesterol; TC, total cholesterol; TG, triglycerides.

**Table 4:** Comparison of MetS components among metabotype risk clusters.

	Metabotype Risk Clusters			
	Low-Risk	Intermediate-Risk	High-Risk	*p*-Value
N	881	295	78	
Components of MetS				
WC (cm)	90.03 ± 0.87	92.15 ± 0.69	93.98 ± 0.75	<0.001
TG (mg/dL)	122.14 ± 2.15	206.02 ± 8.90	215.78 ± 20.38	<0.001
HDLc (mg/dL)	48.43 ± 0.46	46.93 ± 0.78	44.45 ± 1.76	0.039
SBP (mmHg)	125.45 ± 0.57	128.32 ± 0.49	129.87 ± 0.65	0.002
DBP (mmHg)	71.38 ± 0.62	74.34 ± 0.56	74.63 ± 0.67	<0.001
FBG (mg/dL)	121.97 ± 0.81	133.15 ± 1.75	235.00 ± 5.83	<0.001
MetS (*n*, %)				
Yes	434 (49.3)	209 (70.9)	66 (84.6)	<0.001
No	447 (50.7)	86 (29.2)	12 (15.4)	
Number of MetS components met (*n*, %)				
Exactly three components	262 (29.7)	95 (32.2)	25 (32.1)	<0.001
Four components	133 (15.1)	86 (29.2)	26 (33.3)	
All five components	39 (4.4)	28 (9.5)	15 (19.2)	

DBP, diastolic blood pressure; FBG, fasting blood glucose; HDLc, high-density lipoprotein cholesterol; SBP, systolic blood pressure; TG, triglycerides; WC, waist circumference.

**Table 5:** Association between metabotype risk clusters and MetS.

	Metabotype Risk Clusters		
	Low-Risk	Intermediate-Risk	High-Risk
MetS			
Prevalence (*n*, %)	434 (49.3)	209 (70.9)	66 (84.6)
Crude OR (95% CI)	1.0 (ref)	2.50 (1.89–3.32)	5.67 (3.02–10.63)
*p*-value		<0.001	<0.001
Multivariable OR (95% CI)	1.0 (ref)	2.43 (1.82–3.23)	5.46 (2.89–10.30)
*p*-value		<0.001	<0.001
Elevated WC			
Prevalence (*n*, %)	546 (62.0)	192 (65.1)	62 (79.5)
Crude OR (95% CI)	1.0 (ref)	1.14 (0.87–1.51)	2.38 (1.35–4.19)
*p*-value		0.34	0.003
Multivariable OR (95% CI)	1.0 (ref)	1.10 (0.83–1.46)	2.19 (1.24–3.88)
*p*-value		0.49	0.007
Elevated TG			
Prevalence (*n*, %)	212 (24.1)	174 (59.0)	49 (62.8)
Crude OR (95% CI)	1.0 (ref)	4.54 (3.43–5.99)	5.33 (3.29–8.66)
*p*-value		<0.001	<0.001
Multivariable OR (95% CI)	1.0 (ref)	4.38 (3.30–5.81)	4.84 (2.95–7.93)
*p*-value		<0.001	<0.001
Reduced HDLc			
Prevalence (*n*, %)	367 (41.7)	131 (44.4)	46 (59.0)
Crude OR (95% CI)	1.0 (ref)	1.12 (0.86–1.46)	2.01 (1.26–3.22)
*p*-value		0.41	0.004
Multivariable OR (95% CI)	1.0 (ref)	1.13 (0.86–1.49)	2.24 (1.37–3.65)
*p*-value		0.37	0.001
Elevated BP			
Prevalence (*n*, %)	374 (42.5)	148 (50.2)	40 (51.3)
Crude OR (95% CI)	1.0 (ref)	1.37 (1.05–1.78)	1.43 (0.90–2.27)
*p*-value		0.021	0.13
Multivariable OR (95% CI)	1.0 (ref)	1.42 (1.09–1.86)	1.60 (0.99–2.56)
*p*-value		0.010	0.05
Elevated FBG			
Prevalence (*n*, %)	759 (86.2)	266 (90.2)	78 (100.0)
Crude OR (95% CI)	1.0 (ref)	1.47 (0.96–2.26)	-
*p*-value		0.08	-
Multivariable OR (95% CI)	1.0 (ref)	1.40 (0.91–2.16)	-
*p*-value		0.13	-

BP, blood pressure; FBG, fasting blood glucose; HDLc, high-density lipoprotein cholesterol; Ref, reference; TG, triglycerides; WC, waist circumference. The multivariable models were adjusted for sex, age, alcohol consumption, smoking status, physical activity (both vigorous and moderate intensity), and total energy intake.

## Data Availability

The original data presented in the study are openly available from the Korea Disease Control and Prevention Agency (KDCA) through the Korea National Health and Nutrition Examination Survey (KNHANES) website (https://knhanes.kdca.go.kr/knhanes, accessed on 18 March 2025). Researchers may access the data after a simple registration process and agreement to the data use policy.

## Supplementary Materials

The following supporting information can be downloaded at https://www.mdpi.com/article/10.3390/diseases13080239/s1, Figure S1: Elbow plot showing within-cluster sum of squares (WCSS) for k = 2 to 6; Figure S2: PCA of metabotype risk clusters; Table S1: Diagnostic criteria for metabolic syndrome by different organizations; Table S2: Sociodemographic and lifestyle characteristics across metabotype risk clusters by sex; Table S3: Comparison of metabolic biomarkers across metabotype risk clusters by sex; Table S4: Comparison of MetS components among metabotype risk clusters by sex; Table S5: Association between metabotype risk clusters and MetS by sex.
