# Supplementary material for: Metabotype Risk Clustering Based on Metabolic Disease Biomarkers and Its Association with Metabolic Syndrome in Korean Adults: Findings from the 2016–2023 Korea National Health and Nutrition Examination Survey (KNHANES)
Source: Diseases. 2025 Jul 28;13(8):239. doi: 10.3390/diseases13080239 (PMC12386031; doi:10.3390/diseases13080239)
Supplement: Supplementary file 1 [file diseases-13-00239-s001.zip › diseases-3725436-supplementary.pdf]

Table S1. Diagnostic criteria for metabolic syndrome by different organizations

Component	IDF (2005)	NCEP-ATP III (2001, revised 2005)	WHO (1999)	Korean-Specific Criteria (Modified NCEP-ATP III)
Diagnosis Requirement	Central obesity plus any 2 of the other 4 components	Any 3 of the 5 components	Insulin resistance or diabetes plus any 2 of the other criteria	Any 3 of the 5 components
Central Obesity	WC ≥90 cm (M), ≥80 cm (W)	WC ≥102 cm (M), ≥88 cm (W)	WHR >0.90 (M), >0.85 (W) or BMI >30 kg/m² or WC criteria	WC ≥90 cm (men), ≥85 cm (women) (Korean cut-points)
TG	≥150 mg/dL or treatment	≥150 mg/dL or treatment	≥150 mg/dL or HDL <35 mg/dL (M), <39 mg/dL (W)	≥150 mg/dL or treatment
HDLc	<40 mg/dL (M), <50 mg/dL (W) or treatment	<40 mg/dL (M), <50 mg/dL (W) or treatment	<35 mg/dL (M), <39 mg/dL (W)	<40 mg/dL (men), <50 mg/dL (women) or treatment
BP	Systolic BP ≥130 mmHg or diastolic BP ≥85 mmHg or treatment	Systolic BP ≥130 mmHg or diastolic BP ≥85 mmHg or treatment	Systolic BP ≥140 mmHg or diastolic BP ≥90 mmHg or treatment	Systolic BP ≥130 mmHg or diastolic BP ≥85 mmHg or treatment
FBG	≥100 mg/dL or previously diagnosed type 2 diabetes	≥100 mg/dL or drug treatment for elevated glucose	IGT, IFG, or diabetes	≥100 mg/dL or previously diagnosed type 2 diabetes

BP, blood pressure; FBG, fasting blood glucose; TG, triglycerides; IFG, impaired fasting glucose; IGT, impaired glucose tolerance; M, men; HDLc, high-density lipoprotein cholesterol; WC, waist circumference; W, women.

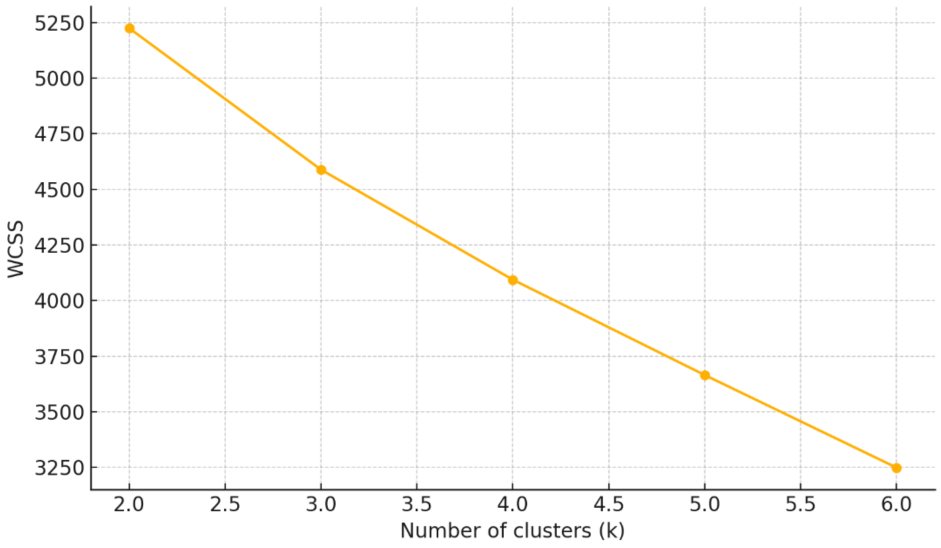

Figure S1. Elbow plot showing within-cluster sum of squares (WCSS) for k = 2 to 6

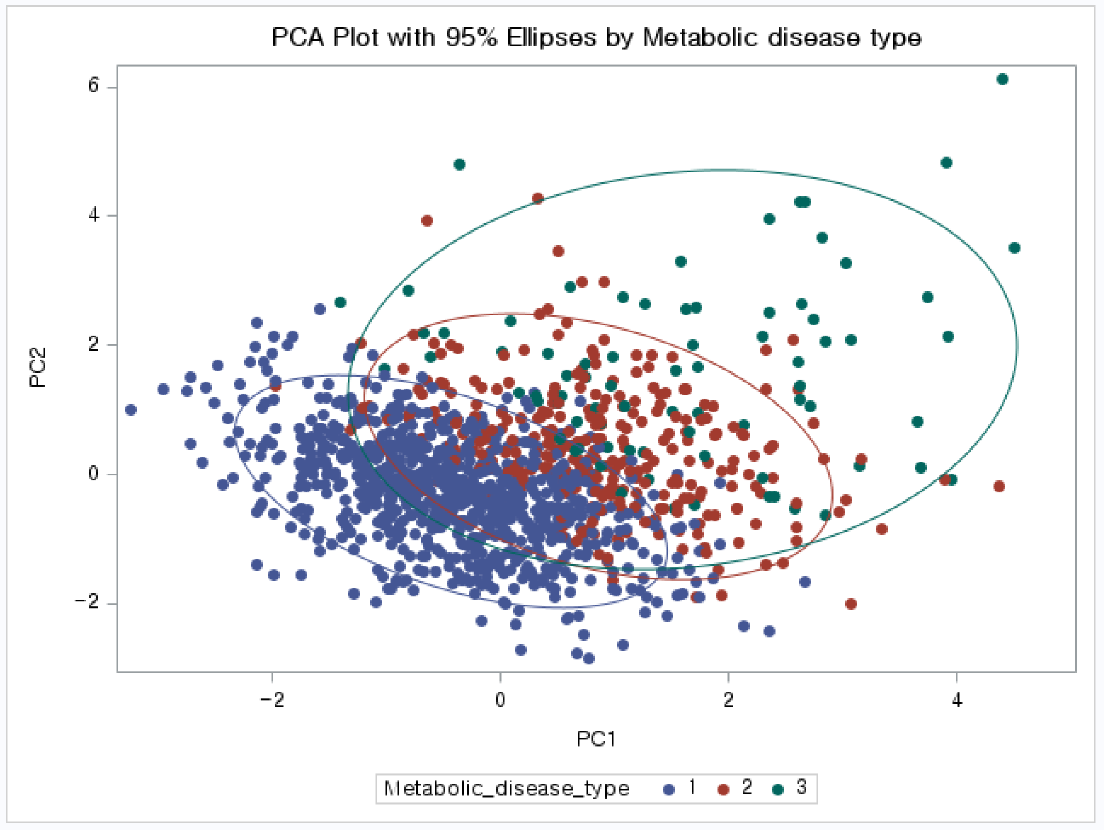

Figure S2. PCA of metabotype risk clusters

Table S2. Sociodemographic and lifestyle characteristics across metabotype risk clusters by sex

	**Men (n = 571)**				**Women (n = 683)**			
	**Low-risk**	**Intermediate-risk**	**High-risk**	***P*-value**	**Low-risk**	**Intermediate-risk**	**High-risk**	***p* - value**
N	398	131	42		483	164	36	
Age (years)	63.73 ± 0.76	60.50 ± 0.96	57.84 ± 2.16	0.004	67.18 ± 0.45	64.39 ± 0.95	60.47 ± 1.71	<0.001
Education (n, %)								
Less than middle school	181 (39.74)	47 (34.65)	17 (35.56)	0.38	357 (70.24)	108 (59.29)	25 (59.15)	0.29
High school	133 (33.45)	45 (29.81)	17 (42.22)		87 (20.44)	39 (24.78)	8 (29.31)	
Over college	84 (26.82)	39 (35.54)	8 (22.22)		39 (9.32)	17 (15.93)	3 (11.54)	
Occupation (n, %)								
Professionals, administrative, management, office jobs	54 (17.71)	21 (17.55)	6 (13.55)	0.84	18 (3.95)	9 (9.33)	1 (2.66)	0.018
Sales and service positions	21 (6.87)	9 (9.26)	4 (10.36)		41 (9.15)	18 (12.04)	7 (25.79)	
Agriculture, manufacturing, mining, army service	140 (31.16)	43 (32.54)	15 (40.97)		104 (19.25)	26 (14.41)	4 (6.63)	
Housekeeping, unemployment, and others	183 (44.27)	58 (40.66)	17 (35.12)		320 (67.65)	111 (64.21)	24 (64.92)	
Alcohol consumption (n, %)								
Past or current	386 (97.11)	122 (91.80)	39 (95.98)	0.031	333 (69.73)	118 (73.34)	27 (82.79)	0.26
Never	12 (2.89)	9 (8.20)	3 (4.02)		150 (30.27)	46 (26.66)	9 (17.21)	
Smoking (n, %)								
Past or current	337 (85.96)	113 (86.34)	30 (63.63)	0.003	38 (7.29)	21 (12.38)	7 (13.34)	0.11
Never	61 (14.04)	18 (13.66)	12 (36.37)		445 (92.71)	143 (87.62)	29 (86.66)	
Vigorous intensity of physical activity (n, %)								
Yes	13 (3.81)	9 (7.07)	1 (2.82)	0.35	4 (0.98)	2 (2.12)	-	0.57
No	385 (96.19)	122 (92.93)	41 (97.18)		479 (99.02)	162 (97.88)	36 (100.0)	
Moderate intensity of physical activity (n, %)								
Yes	79 (22.89)	38 (29.28)	6 (23.59)	0.49	65 (13.52)	17 (12.52)	4 (14.59)	0.96
No	319 (77.11)	93 (70.72)	36 (76.41)		418 (86.48)	147 (87.48)	32 (85.41)	
Antihypertensive medication use (n, %)								
Yes	369 (92.7)	121 (92.4)	38 (90.5)	0.87	467 (96.7)	148 (90.2)	35 (97.2)	0.003
No	29 (7.3)	10 (7.6)	4 (9.5)		16 (3.3)	16 (9.8)	1 (2.8)	
Lipid-lowering medication use (n, %)								
Yes	348 (87.4)	100 (76.3)	32 (76.2)	0.004	465 (96.3)	120 (73.2)	30 (83.3)	<0.001
No	50 (12.6)	31 (23.7)	10 (23.8)		18 (3.7)	44 (26.8)	6 (16.7)	
Antidiabetic medication use (n, %)								
Yes	384 (96.5)	121 (92.4)	41 (97.6)	0.11	463 (95.9)	146 (89.0)	36 (100)	0.001
No	14 (3.5)	10 (7.6)	1 (2.4)		20 (4.1)	18 (11.0)	0 (0.0)	
Obesity (n, %)								
Underweight	4 (0.69)	-	-	0.40	4 (0.53)	-	-	0.24
Normal	91 (21.94)	24 (14.92)	7 (11.36)		119 (24.49)	36 (19.41)	4 (6.83)	
Pre-obese	109 (25.87)	37 (28.81)	13 (33.06)		136 (28.68)	47 (27.93)	14 (41.52)	
Obese	194 (51.50)	70 (56.26)	22 (55.58)		224 (46.31)	81 (52.66)	18 (51.64)	
Total energy intake (kcal/day)	2,004.10	1,944.53	2,365.64	0.46	1,421.56	1,535.16	1,612.87	0.06

	± 62.22	± 82.32	± 339.51		± 26.84	± 55.53	± 102.82	

Values for categorical variables are shown as number and percentage (n, %), and continuous variables as mean ± SE, adjusted for the complex sampling design. A *p*-value below 0.05 was considered significant.

Table S3. Comparison of metabolic biomarkers across metabotype risk clusters by sex

	**Men**				**Women**			
	**Low-risk**	**Intermediate-risk**	**High-risk**	***P*-value**	**Low-risk**	**Intermediate-risk**	**High-risk**	***p* -value**
Biomarkers used for metabotype clusters								
BMI (kg/m^2)	25.78 ± 0.26	26.38 ± 0.34	27.61 ± 0.75	0.044	25.57 ± 0.19	26.56 ± 0.35	26.55 ± 0.64	0.025
Uric acid (mg/dL)	5.46 ± 0.09	5.71 ± 0.14	5.56 ± 0.23	0.33	4.58 ± 0.06	5.00 ± 0.10	4.47 ± 0.24	<0.001
FBG (mg/dL)	123.67 ± 1.10	132.11 ± 2.44	232.23 ± 7.29	<0.001	120.21 ± 1.17	134.25 ± 2.46	239.20 ± 9.22	<0.001
HDLc (mg/dL)	46.17 ± 0.64	44.75 ± 1.16	41.03 ± 1.91	0.040	50.78 ± 0.62	49.24 ± 0.99	49.65 ± 2.47	0.41
Non-HDLc (mg/dL)	91.24 ± 1.09	153.01 ± 2.76	115.20 ± 6.16	<0.001	92.87 ± 0.86	155.97 ± 2.58	126.08 ± 8.09	<0.001
Other biomarkers								
HbA1c (%)	6.80 ± 0.04	7.06 ± 0.11	9.46 ± 0.30	<0.001	6.82 ± 0.05	7.28 ± 0.11	10.00 ± 0.34	<0.001
TC (mg/dL)	137.42 ± 1.25	197.77 ± 3.18	156.27 ± 6.51	<0.001	143.67 ± 1.05	205.26 ± 2.93	175.76 ± 7.12	<0.001
TG (mg/dL)	124.72 ± 3.06	237.92 ± 14.02	239.95 ± 27.93	<0.001	119.46 ± 2.95	172.31 ± 8.77	179.02 ± 20.51	<0.001
AST (IU/L)	26.63 ± 0.64	26.83 ± 1.32	26.74 ± 2.09	0.98	25.72 ± 0.55	26.34 ± 1.30	29.25 ± 3.85	0.61
ALT (IU/L)	28.14 ± 0.97	27.90 ± 1.46	30.88 ± 2.94	0.65	24.48 ± 0.79	25.52 ± 1.80	33.49 ± 5.90	0.29
BUN (mg/dL)	17.25 ± 0.31	17.81 ± 0.76	17.46 ± 0.93	0.79	16.96 ± 0.29	16.45 ± 0.56	16.65 ± 0.94	0.72
Creatinine (mg/dL)	1.07 ± 0.02	1.11 ± 0.05	1.03 ± 0.02	0.27	1.00 ± 0.01	1.03 ± 0.03	1.01 ± 0.04	0.51
Hemoglobin (g/dL)	14.50 ± 0.08	14.63 ± 0.16	15.05 ± 0.26	0.13	12.96 ± 0.06	13.43 ± 0.10	13.74 ± 0.20	<0.001
Hematocrit (%)	43.54 ± 0.24	44.00 ± 0.41	44.56 ± 0.64	0.28	39.54 ± 0.19	40.93 ± 0.26	41.46 ± 0.63	<0.001

ALT, alanine aminotransferase; AST, aspartate aminotransferase; BMI, body mass index; BUN, blood urea nitrogen; FBG, fasting blood glucose; HbA1c, hemoglobin A1c; HDLc, high-density lipoprotein cholesterol; non-HDLc, non-high-density lipoprotein cholesterol; TC, total cholesterol; TG, triglyceride.

Table S4. Comparison of MetS components among metabotype risk clusters by sex

	Men				Women			
	Low-risk	Intermediate-risk	High-risk	*P*-value	Low-risk	Intermediate-risk	High-risk	*p* -value
Components of MetS								
WC (cm)	92.54 ± 8.84	93.95 ± 9.30	95.64 ± 9.49	0.05	87.82 ± 8.83	89.70 ± 8.85	91.41 ± 8.44	0.008
TG (mg/dL)	124.72 ± 3.06	237.92 ± 14.02	239.95 ± 27.93	<0.001	119.46 ± 2.95	172.31 ± 8.77	179.02 ± 20.51	<0.001
HDLc (mg/dL)	46.17 ± 0.64	44.75 ± 1.16	41.03 ± 1.91	0.040	50.78 ± 0.62	49.24 ± 0.99	49.65 ± 2.47	0.41
SBP (mmHg)	124.71 ± 14.09	128.47 ± 13.85	130.07 ± 16.86	0.005	128.19 ± 15.82	130.70 ± 17.09	130.42 ± 18.12	0.20
DBP (mmHg)	73.11 ± 9.07	76.15 ± 10.05	78.74 ± 11.65	<0.001	72.54 ± 8.59	74.34 ± 8.87	72.31 ± 10.44	0.07
FBG (mg/dL)	123.67 ± 1.10	132.11 ± 2.44	232.23 ± 7.29	<0.001	120.21 ± 1.17	134.25 ± 2.46	239.20 ± 9.22	<0.001
MetS (*n*, %)								
Yes	172 (43.2)	92 (70.2)	37 (88.1)	<0.001	262 (54.2)	117 (71.3)	29 (80.6)	<0.001
No	226 (56.8)	39 (29.8)	5 (11.9)		221 (45.8)	47 (28.7)	7 (19.4)	
Number of MetS components met (*n*, %)								
Exactly 3 components	112 (28.1)	49 (37.4)	14 (33.3)	0.009	150 (31.1)	46 (28.1)	11 (30.6)	0.006
4 components	48 (12.1)	34 (26.0)	15 (35.7)		85 (17.6)	52 (31.7)	11 (30.6)	
All 5 components	12 (3.0)	9 (6.9)	8 (19.1)		27 (5.6)	19 (11.6)	7 (19.4)	

DBP, diastolic blood pressure; FBG, fasting blood glucose; HDLc, high-density lipoprotein cholesterol; SBP, systolic blood pressure; TG, triglyceride; WC, waist circumference.

Table S5. Association between metabotype risk clusters and MetS by sex

	Men			Women		
	**Low-risk**	**Intermediate-risk**	**High-risk**	**Low-risk**	**Intermediate-risk**	**High-risk**
MetS						
Prevalence (*n*, %)	172 (43.2)	92 (70.2)	37 (88.1)	262 (54.2)	117 (71.3)	29 (80.6)
Crude OR (95% CI)	1.0 (ref)	3.10 (2.03-4.74)	9.72 (3.74-25.26)	1.0 (ref)	2.10 (1.43-3.08)	3.49 (1.50-8.13)
p -value		<0.001	<0.001		<0.001	0.004
Multivariable OR (95% CI)	1.0 (ref)	2.89 (1.87-4.47)	9.22 (3.49-24.36)	1.0 (ref)	2.09 (1.42-3.08)	3.70 (1.56-8.75)
p -value		<0.001	<0.001		<0.001	0.003
Elevated WC						
Prevalence (*n*, %)	245 (61.6)	83 (63.4)	33 (78.6)	301 (62.3)	109 (66.5)	29 (80.6)
Crude OR (95% CI)	1.0 (ref)	1.08 (0.72-1.63)	2.29 (1.07-4.92)	1.0 (ref)	1.20 (0.83-1.74)	2.51 (1.08-5.84)
p -value		0.71	0.034		0.34	0.033
Multivariable OR (95% CI)	1.0 (ref)	1.01 (0.66-1.53)	2.13 (0.97-4.65)	1.0 (ref)	1.14 (0.78-1.67)	2.36 (1.00-5.57)
p -value		0.97	0.06		0.49	0.05
Elevated TG						
Prevalence (*n*, %)	100 (25.1)	86 (65.7)	28 (66.7)	112 (23.2)	88 (53.7)	21 (58.3)
Crude OR (95% CI)	1.0 (ref)	5.60 (3.72-8.72)	5.96 (3.02-11.77)	1.0 (ref)	3.84 (2.64-5.57)	4.64 (2.31-9.30)
p -value		<0.001	<0.001		<0.001	<0.001
Multivariable OR (95% CI)	1.0 (ref)	5.41 (3.49-8.39)	5.80 (2.87-11.74)	1.0 (ref)	3.69 (2.54-5.38)	4.16 (2.05-8.44)
p -value		<0.001	<0.001		<0.001	<0.001
Reduced HDLc						
Prevalence (*n*, %)	121 (30.4)	38 (29.0)	25 (59.5)	246 (50.9)	93 (56.7)	21 (58.3)
Crude OR (95% CI)	1.0 (ref)	0.94 (0.61-1.44)	3.37 (1.75-6.46)	1.0 (ref)	1.26 (0.88-1.80)	1.35 (0.68-2.68)
p -value		0.76	<0.001		0.20	0.39
Multivariable OR (95% CI)	1.0 (ref)	0.89 (0.57-1.40)	3.27 (1.67-6.42)	1.0 (ref)	1.30 (0.91-1.87)	1.41 (0.70-2.84)
p -value		0.62	<0.001		0.15	0.33
Elevated BP						
Prevalence (*n*, %)	151 (37.9)	67 (51.2)	23 (54.8)	223 (46.2)	81 (49.4)	17 (47.2)
Crude OR (95% CI)	1.0 (ref)	1.71 (1.15-2.55)	1.98 (1.04-3.76)	1.0 (ref)	1.14 (0.80-1.62)	1.04 (0.53-2.06)
p -value		0.008	0.037		0.48	0.90
Multivariable OR (95% CI)	1.0 (ref)	1.75 (1.17-2.63)	2.17 (1.12-4.19)	1.0 (ref)	1.19 (0.83-1.71)	1.28 (0.63-2.58)
p -value		0.007	0.021		0.34	0.50
Elevated FBG						
Prevalence (*n*, %)	348 (87.4)	118 (90.1)	42 (100.0)	411 (85.1)	148 (90.2)	36 (100.0)
Crude OR (95% CI)	1.0 (ref)	1.30 (0.68-2.49)	-	1.0 (ref)	1.62 (0.91-2.88)	-
p -value		0.42	-		0.10	-
Multivariable OR (95% CI)	1.0 (ref)	1.09 (0.57-2.11)	-	1.0 (ref)	1.58 (0.88-2.81)	-

p -value	0.79	-	0.12	-

BP, blood pressure; FBG, fasting blood glucose; HDLc, high-density lipoprotein cholesterol; Ref, reference; TG, triglyceride; WC, waist circumference. The multivariable models were adjusted for age, alcohol consumption, smoking status, physical activity (both vigorous and moderate intensity), and total energy intake.
